# Epitranscriptomics: Correlation of N^6^-methyladenosine RNA methylation and pathway dysregulation in the hippocampus of HIV transgenic rats

**DOI:** 10.1371/journal.pone.0203566

**Published:** 2019-01-17

**Authors:** Yu Fu, Barry Zorman, Pavel Sumazin, Pietro Paolo Sanna, Vez Repunte-Canonigo

**Affiliations:** 1 Department of Immunology and Microbiology and Department of Neuroscience, The Scripps Research Institute, La Jolla, CA, United States of America; 2 Department of Pediatrics, Dan L. Duncan Cancer Center, Baylor College of Medicine, Houston, TX, United States of America; Florida Atlantic University, UNITED STATES

## Abstract

Internal RNA modifications have been known for decades, however their roles in mRNA regulation have only recently started to be elucidated. Here we investigated the most abundant mRNA modification, N^6^-methyladenosine (m^6^A) in transcripts from the hippocampus of HIV transgenic (Tg) rats. The distribution of m^6^A peaks within HIV transcripts in HIV Tg rats largely corresponded to the ones observed for HIV transcripts in cell lines and T cells. Host transcripts were found to be differentially m^6^A methylated in HIV Tg rats. The functional roles of the differentially m^6^A methylated pathways in HIV Tg rats is consistent with a key role of RNA methylation in the regulation of the brain transcriptome in chronic HIV disease. In particular, host transcripts show significant differential m^6^A methylation of genes involved in several pathways related to neural function, suggestive of synaptodendritic injury and neurodegeneration, inflammation and immune response, as well as RNA processing and metabolism, such as splicing. Changes in m^6^A methylation were usually positively correlated with differential expression, while differential m^6^A methylation of pathways involved in RNA processing were more likely to be negatively correlated with gene expression changes. Thus, sets of differentially m^6^A methylated, functionally-related transcripts appear to be involved in coordinated transcriptional responses in the context of chronic HIV. Altogether, our results support that m^6^A methylation represents an additional layer of regulation of HIV and host gene expression *in vivo* that contributes significantly to the transcriptional effects of chronic HIV.

## Introduction

An extensive repertoire of modifications is believed to contribute to the regulation of RNA processing, metabolism and expression. It has long been known that transfer RNA (tRNA), ribosomal RNA (rRNA) and both mRNA and noncoding RNA (ncRNA) contain multiple modifications [1–5]. At least 10 distinct modified bases have now been identified in mammalian mRNAs, suggesting the existence of an “epitranscriptome” [6–9]. In addition to the 7-methylguanosine cap that is added at the 5’ end of all cellular mRNAs, several internal RNA modifications have been described, which include N^6^-methyladenosine (m^6^A), 2’-O-methyladenosine (Am), N^6^-2’-O-methyladenosine (m^6^Am), pseudouridine, and 5-methylcytosine, among others [6–10]. Of these, by far the most abundant RNA modification identified is N^6^-methyladenosine (m^6^A) [1, 2]. The occurrence of m^6^A in polyadenylated RNA was originally reported in 1974 [11, 12]. While m^6^A has been found in mRNAs from diverse tissue types, the brain has especially high levels of m^6^A [13]. mRNAs are modified by m^6^A preferentially in the 3′ untranslated regions (UTRs), near the stop codons within mRNAs and within long internal exons, and m^6^A modification appears to be conserved between humans and rodents [1].

Reminiscent of DNA epigenetics, the occurrence of both stable and dynamically regulated m^6^A-modified RNA sites has been suggested [1, 2, 14–16]. mRNAs encoding housekeeping genes are less likely to be m^6^A methylated, while highly regulated mRNAs often contain m^6^A residues [8, 10]. RNA is m^6^A methylated within the consensus motifs G(m^6^A)C and A(m^6^A)C [17]. RNA modification by m^6^A appears to contribute to the regulation of gene expression and is increasingly being implicated in disease. Evidence suggests that RNA m^6^A methylation contributes to the regulation of splicing, nuclear RNA export, mRNA stability, and translation [18–23]. RNA m^6^A methylation is at least in part dynamically regulated and potentially reversible [6–8]. Multiple proteins are involved in the regulation of m^6^A RNA methylation and the fate of m^6^A methylated RNA [6–8]. The fat mass and obesity associated gene (FTO), a m^6^A demethylase, has been linked to a variety of biological processes including dopaminergic neuron regulation [24] and a variety of human diseases including obesity, type 2 diabetes (T2DM), cancer, attention-deficit/hyperactivity disorder, and Alzheimer's disease [25–31]. The m^6^A contents in the RNAs of T2DM patients and diabetic rats are significantly lower than controls, while the level of FTO in T2DM patients was significantly higher [31]. Loss of ALKBH5, the other known mammalian demethylase, is associated with increased m^6^A content in mRNA and impaired fertility [32].

Multiple viral RNAs have long been known to be m^6^A modified. Classic studies by Shatkin and associates in the 70s showed specific patterns of m^6^A modification in transcripts from both DNA and RNA viruses such as influenza virus, adenovirus, papovavirus, and flavivirus, among others [3, 7, 33–35]. Retrovirus transcripts have also been found to be m^6^A modified [36, 37]. The phenotypic consequences of these modifications have recently begun to emerge and involve regulation of viral expression and infectivity, reviewed in [7, 38–41]. Recent studies showed m^6^A modification in HIV-1 transcripts contributes to regulate HIV-1 mRNA *in vitro* [42–44]. Another possible role of m^6^A modification of viral RNAs appears to be antiviral innate immune evasion, since the presence of m^6^A on viral RNA has been shown to reduce its activation of toll-like receptor 3 signaling [45].

Here we investigated m^6^A methylation of RNA in the hippocampus of HIV Tg rats. HIV-1 Tg rats harbor a non-replicating HIV-1 transgene and express multiple HIV-1 proteins under the viral LTR in macrophages, lymphocytes and disease-relevant glial cells, such as microglia and astrocytes, but not in neurons [46, 47]. HIV-1 Tg rats exhibit deficits in working memory [47], spatial [48] and reversal learning [49, 50], before the onset of motor deficits [47–49]. This is reminiscent of HAND in the cART era, which primarily impacts learning and executive functioning in a shift from the prominent slowed motor and speed of processing deficits in the pre-cART era [51].

We observed that distribution of peaks of m^6^A RNA methylation in HIV transcripts in HIV Tg rats largely corresponds to the ones reported for HIV transcripts in cell lines and T cells *in vitro* [42–44]. Further, we observed that host genes in HIV Tg rats show significant differential m^6^A methylation of genes involved in several pathways related to neural function, suggestive of synaptodendritic injury and neurodegeneration, consistent with our previous observations in both HIV Tg rats and humans with HIV [52, 53]. Transcripts with significant differential m^6^A methylation and expression are also involved in immune response and inflammation, also consistently with gene expression changes in HIV Tg rats and humans with HIV [52, 53]. We also observed differential m^6^A methylation in transcripts of pathways involved in RNA splicing and processing, which are processes previously implicated in the role of m^6^A RNA methylation [6–10].

## Results

### RNA m^6^A methylation in viral and host transcripts from the hippocampus of HIV Tg rats

**Fig 1:**
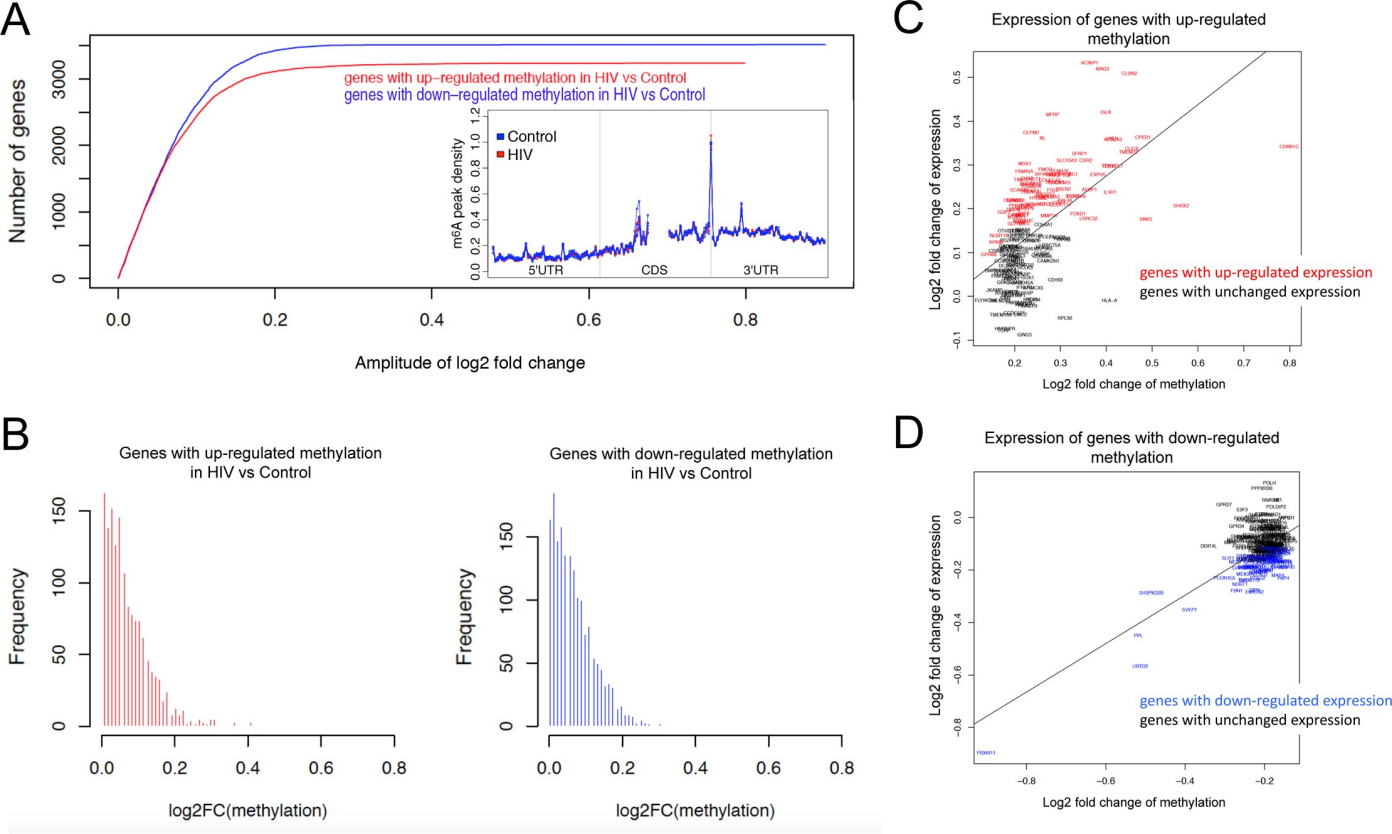
Distribution of genes with differential m^6^A RNA methylation in the hippocampus of HIV Tg rats. **A)** A larger number of genes with down-regulation of m^6^A methylation was observed in HIV Tg rats than the number of genes with up-regulated methylation, in particular, 201 genes with significant down-regulated methylation (p-value<0.05) while 172 genes were found with significant up-regulated methylation (p-value<0.05). Inset: Distribution of m^6^A peaks within hippocampal mRNAs of HIV Tg and control rat. Transcript architecture is shown underneath: 5’ untranslated region (UTR); coding sequence (CDS); and 3’ UTR. The density of m^6^A peaks was greater on the distal CDS and 3’UTR and showed the greatest enrichment in the immediate vicinity of the stop codon at the CDS-3’UTR boundary. **B) The distribution of** log2 fold change (log2FC) of genes with up-regulated and down-regulated methylation **C,D)** Correlation between the methylation and expression changes for genes with up-regulated and down-regulated methylation (Pearson correlation = 0.67 and 0.58 respectively). Among the 201 genes with down-regulated methylation, the expression of 127 genes are also down-regulated while the rest remain unchanged; Among the 172 genes with up-regulated methylation, the expression of 83 genes are also up-regulated while expression of 89 genes remain unchanged.

We carried out MeRIP from poly-A selected RNA extracted from the hippocampus of HIV Tg and control rats using an antibody specific for m^6^A. As shown in Fig 1A and 1B, the number of transcripts with significant increased and significant decreased m^6^A methylation was similar, although a slightly larger number of genes with down-regulation of m^6^A methylation was observed than genes with up-regulated methylation in the hippocampus of HIV Tg rats (201 genes with down-regulated methylation and 172 genes with significant up-regulated methylation; p-value<0.05, S1 Table). Analysis of the distribution of m^6^A peaks within host mRNAs in HIV Tg and control rat showed that m^6^A peaks were enriched (fold change of average coverage equals to 2.3) in the distal CDS and 3’UTR of the mRNAs and especially in the immediate vicinity of the stop codon (Fig 1A). Differential m^6^A methylation in poly-A selected transcripts mostly showed either positive correlated changes in m^6^A and mRNA levels or were not differentially expressed (Fig 1C and 1D).

**Fig 2:**
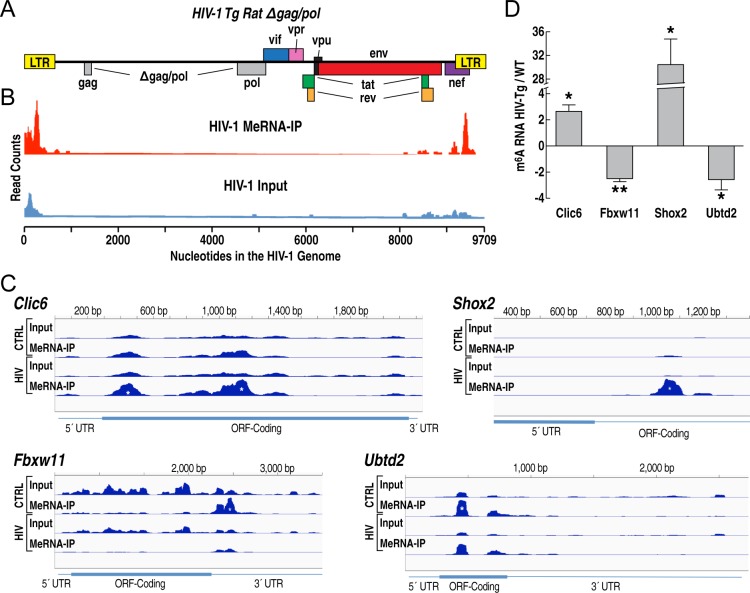
Pattern of m^6^A methylation in HIV and host transcripts of HIV Tg rats. **A)** Outline of HIV genome organization and gag/pol deletion in HIV Tg rats. **B)** m^6^A methylation in HIV RNA from HIV Tg rats (HIV-1 MeRNA-IP) is enriched in transcripts corresponding to the 5’ and 3’ of the virus with a similar overall distribution as observed in HIV transcripts *in vitro* [42–44]; signal obtained from RNA-Seq of input RNA from the hippocampus of HIV Tg rats (HIV-1 Input). **C)** Distribution of m^6^A methylation in representative host genes with significant differential m^6^A methylation (*significantly differentially methylated peaks). **D)** PCR validation of significantly differentially methylated intervals in the genes in D (*p<0.05, *p<0.01 by *t* test).

We then investigated the distribution of m^6^A modification in HIV-1 transcripts in HIV Tg rats (Fig 2). The HIV-1 Tg rats used in the present study harbor a gag/pol-deleted HIV-1 provirus under the control of the long-terminal repeat (LTR) promoter [46], resulting in the co-expression of multiple HIV-1 proteins in disease-relevant central nervous system (CNS) cells such as microglia and astrocytes, but not in neurons [46, 52, 54]. The organization of the HIV transgene in HIV Tg rats is shown in Fig 2A. The m^6^A methylation in HIV transcripts in the hippocampus of HIV Tg rats showed enrichment in the 5’ and 3’ of the provirus most prominently corresponding to the 5’ and 3’ UTRs and LTRs and the *nef* open reading frame (ORF), which partially overlaps with the 3’ LTR (Fig 2B). The location of this 3’ cluster of m^6^A methylation in the HIV genome can affect most of the virus transcripts [55]. The present distribution of m^6^A methylation is in general overall agreement with the pattern of m^6^A methylation of HIV transcripts in cell lines and lymphocytes acutely infected or transfected with replication-competent HIV-1 [42–44]. In particular, m^6^A RNA methylation corresponding to the *nef* ORF was observed in 3 studies using cell lines and lymphocytes *in vitro* [42–44]. Two of these studies also observed significant m^6^A methylation over the ORF of *rev* and/or *env* genes, among others [42, 44]. This pattern of m^6^A methylation was also seen in *in vitro-*infected CD4^+^ T cell lines and primary human CD4^+^ T cells [42, 44]. However, a previous study did not detect m^6^A methylation signal at the 5’ of the virus transcript in *in vitro*-infected human CD4^+^ CEM-SS T cell line [43]. RT-PCR validation of host genes with differential m^6^A methylation between HIV Tg and wild-type rats are shown in Fig 2C and 2D. Altogether, these results show that both HIV and host transcripts are differentially m^6^A methylated in HIV Tg rats.

### Pathway analysis of differential m^6^A RNA methylated genes in the hippocampus of HIV Tg rats

**Fig 3:**
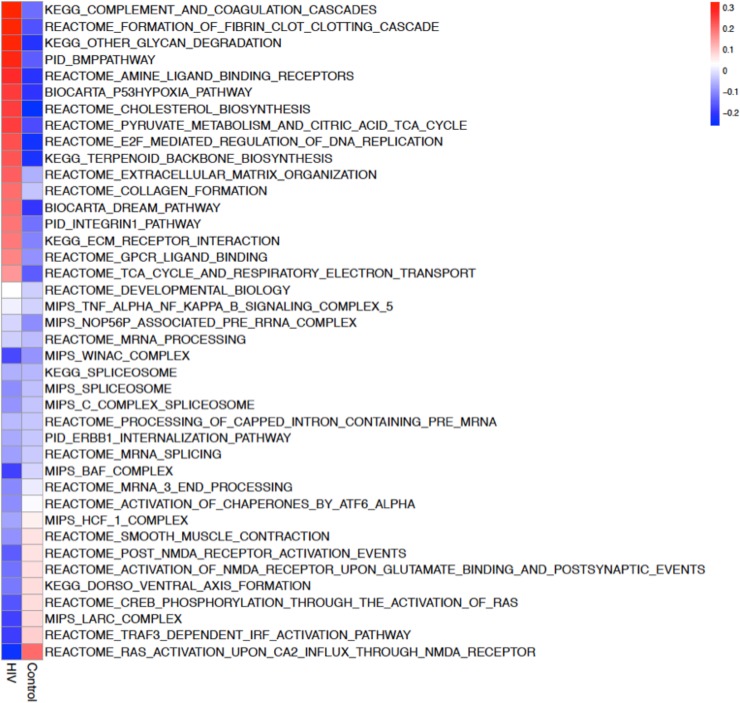
Pathway analysis of differential m^6^A RNA methylation in the hippocampus of HIV Tg rats. Top 40 differential m^6^A methylation pathways in the hippocampus of HIV Tg rats by Gene Set Enrichment Analysis (GSEA) (Complete list in S2 Table).

**Fig 4:**
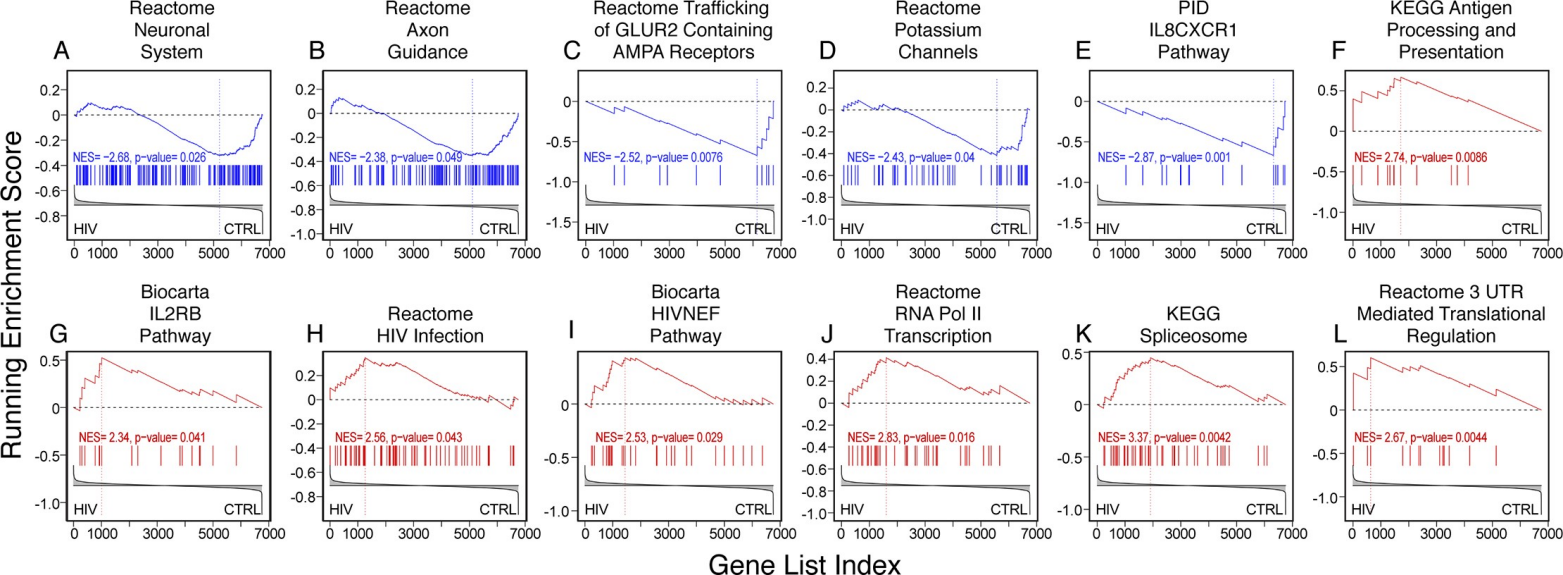
Representative host pathways involving differentially m^6^A methylated genes in HIV Tg rats. **A-D)** Several pathways containing genes differentially m^6^A modified are involved in neural function and are indicative of synaptodendritic injury [57–60], consistent with differential expression of genes in these ontology classes [52, 53]; **E-G)** pathways involved in inflammation and immune response were also differentially m^6^A methylated, consistent with previous observations both in humans and HIV Tg rats [52, 53]; **H,I)** differentially m^6^A methylated gene transcripts also included pathways related to HIV infection; **J-L)** and RNA metabolism and processing, e.g. splicing, which are processes in which m^6^A RNA methylation has been previously implicated [6–10]. Significance is indicated in each plot (Complete list in S2 Table).

We used the Gene Set Enrichment Analysis (GSEA) algorithm [56] to identify molecular pathways that are differentially regulated by m^6^A RNA methylation in HIV-1 Tg rats (Fig 3, S2 Table). A total of 74 significantly differentially regulated pathways was observed (p-value < 0.05, and absolute normalized enriched score (NES) > 2, S2 Table) of which 44 showed increased m^6^A RNA methylation and 30 showed decreased m^6^A methylation (S2 Table). The top 40 differentially regulated pathways are shown in Fig 3. In host genes of HIV Tg rats, significant differential m^6^A methylation was seen in pathways relevant to neural function, suggestive of synaptodendritic injury [57–60], Fig 4. Other transcripts with significant differential m^6^A methylation were identified as involved in inflammation and immune response, also consistently with previous observations both in humans and HIV Tg rats [52, 53], Fig 4. Other genes belong to pathways involved in RNA splicing and processing (Fig 4). However, we did not observe significant differential expression of key transcripts involved in the regulation of m^6^A methylation by RT-PCR including FTO, Mettl3, Alkbh5, Ythdf1, Ythdf2, and Wtap (*p*>0.05 by *t* test; Supplementary Fig 1).

**Fig 5:**
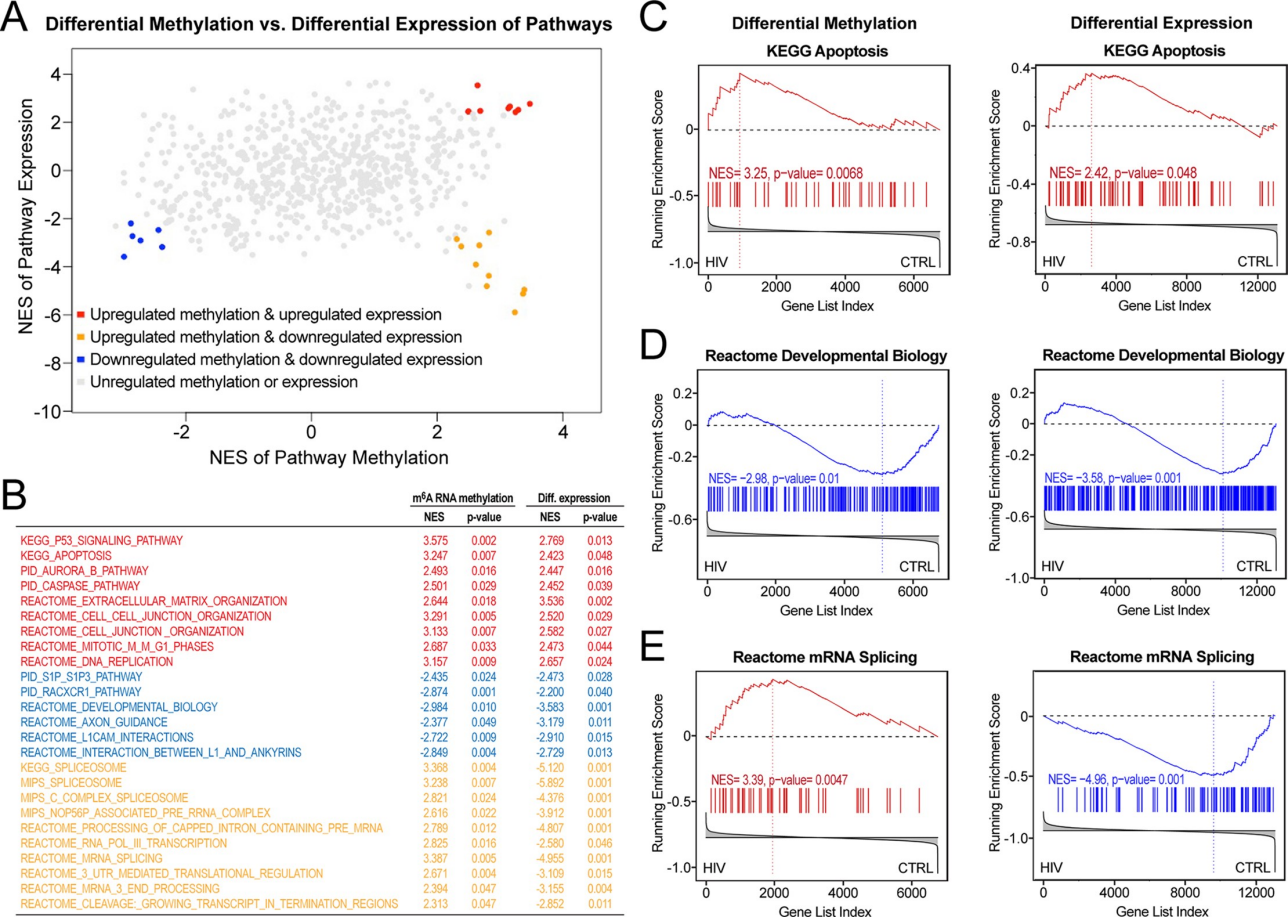
Correlation of m^6^A methylation and gene expression. **A)** The normalized enrichment scores (NES) of pathways showing both significant differential m^6^A RNA methylation (NES of pathway methylation) and gene expression (NES of pathway expression). RNA m^6^A methylation was either directly or inversely correlated with gene expression. Pathways with increased m^6^A RNA methylation and expression are shown in red; pathways with decreased m^6^A RNA methylation and expression are shown in blue; pathways with increased m^6^A RNA methylation and decreased expression are shown in orange. **B)** Pathways showing increased differential m^6^A RNA methylation and expression included some pathways involved in apoptosis and tissue responses (red); pathways showing decreased differential m^6^A RNA methylation and expression included some pathways involved in neuronal trophisms and function (blue); pathways showing increased differential m^6^A RNA methylation and decreased expression included pathways involved in RNA processing and metabolism. **C)** Example of pathway showing increased differential m^6^A RNA methylation and expression, “KEGG Apopotosis”; **D)** Example of pathway showing decreased differential m^6^A RNA methylation and expression, “Reactome Developmental Biology”; **E)** Example of pathway showing increased differential m^6^A RNA methylation and decreased expression, “Reactome mRNA Splicing”.

Representative differentially methylated m^6^A pathways related to neural function include “Reactome Neuronal Systems”, “KEGG Axon Guidance”, and “Reactome Trafficking of Glur2 containing AMPA Receptors” (Fig 4) and “Reactome Developmental Biology” (Fig 5), among others (S2 Table). These pathways are indicative of synaptodendritic injury and reduced trophic support [57–60], and are consistent with differential expression of genes in these ontology classes in both humans with HIV and HIV Tg rats [52, 53]. Among pathways involved in inflammation and immune response, differentially m^6^A methylated pathways are “MIPS TNF-alpha NF Kappa B Signaling Complex”, “PID IFNG Pathway”, “Reactome Class I MHC Mediates Antigen Processing and Presentation”, “PID IL8CXCR1 Pathways” (among others Figs 4 and 5 and S2 Table). Genesets related to HIV infection were also differentially methylated, such as Biocarta HIVNEF Pathway and Reactome HIV Infection (Fig 4). Additional pathways up-regulated in HIV Tg rats were pathways related to apoptosis (Fig 5). These pathways are consistent with previous observations of inflammation and immune dysregulation in both humans and HIV Tg rats [52, 53]. Interestingly, other differentially m^6^A methylated gene transcripts were in pathways involved in RNA splicing, metabolism and processing (Figs 4 and 5). Examples of the latter pathways are “Reactome Pol II Transcription”, Kegg Spliceosome, Reactome 3 UTR Mediated Translational Regulation”, “Reactome mRNA Splicing” (Figs 4 and 5 and S2 Table). These pathways are suggestive of a key role of m^6^A RNA methylation in RNA splicing, metabolism and processing [6–10].

### Correlation of differential m^6^A RNA methylation and expression in pathways of the hippocampus of HIV Tg rats

Next we investigated the correlation of differential m^6^A RNA methylation and expression in pathways of the hippocampus of HIV Tg rats. As shown in Fig 5A, pathways that showed both significant differential m^6^A RNA methylation and expression by GSEA were either directly or inversely correlated with changes in m^6^A RNA methylation and gene expression. In particular, 9 pathways showed both increased m^6^A RNA methylation and transcription (Fig 5A, 5B and 5C); 6 pathways showed both decreased m^6^A RNA methylation and transcription (Fig 5A, 5B and 5D); and 10 pathways showed increased m^6^A RNA methylation but decreased transcription (Fig 5A, 5B and 5E). Pathways showing increased m^6^A RNA methylation and expression included some pathways involved in apoptosis and tissue responses, pathways showing decreased m^6^A RNA methylation and expression included some pathways involved in neuronal trophisms and function, while pathways showing increased m^6^A RNA methylation and decreased expression included pathways involved in RNA processing and metabolism (Fig 5A–5E).

## Discussion

Here we report for the first time patterns of RNA m^6^A methylation, the most abundant internal mRNA modification, *in vivo* in an animal model of HIV. Available data on m^6^A methylation of eukaryotic and HIV transcripts are primarily from *in vitro* studies. In particular, we profiled RNA m^6^A methylation in the hippocampus of HIV Tg rats, a small animal model of concomitant low level expression of multiple HIV-1 products in disease-relevant cells in the CNS [47], which also characterizes HIV-associated neurocognitive disorder (HAND) in the setting of viral suppression.

We found that both HIV and host transcripts of HIV Tg rats contain m^6^A methylation. The distribution of m^6^A peaks within host mRNAs in the hippocampus of HIV Tg and control rat was greater in the distal CDS and 3’UTR of the mRNAs with the greatest density in the immediate vicinity of the stop codon, reminiscent of the distribution of m^6^A peaks observed in cell lines *in vitro* and total brain RNA [1, 2, 61]. The distribution of m^6^A methylation in HIV transcripts of HIV Tg rats largely corresponded to the ones described for HIV transcripts *in vitro* in cell lines and lymphocytes acutely infected or transfected with replication-competent HIV-1 [42–44]. Minor differences with those studies as well as among them likely reflect the acute nature of the infection or transfection used in those studies *vs*. the chronic expressions of HIV transcripts in Tg rats in an *in vivo* setting and different cell lines or lymphocytes used in those studies *vs*. brain parenchyma in the present study where HIV is expressed in microglia and astrocytes [52].

Pathway analysis by GSEA of differential m^6^A RNA methylation and differential mRNA expression showed that pathways affected by m^6^A methylation is consistent with a key role of RNA methylation in the regulation of the brain transcriptome in chronic HIV disease [52, 53]. Host pathways identified by differential m^6^A methylation in the present study involve cellular functions that are key in the pathogenesis of neuroAIDS, such as pathways indicative of synaptodendritic injury, neurodegeneration and apoptosis as well as pathways involved in inflammation and immune response, overall consistent with our previous pathway analyses of both HIV Tg rats and humans with HIV [52, 53]. Additional pathways were involved in RNA splicing and processing, consistent with the emerging roles of m^6^A methylation in these processes [10, 13, 15, 62]. This suggests that m^6^A methylation plays a role as a broad regulator of gene expression in fine-tuning the regulation of host genes in HIV infection.

In the present setting of chronic HIV disease, both positively and negatively regulated differentially m^6^A methylated transcripts were observed. Thus, the present results do not suggest a global, unidirectional change in the level of m^6^A methylation in HIV Tg rats. Rather, GSEA pathway analysis indicates that multiple biological processes are affected by differential m^6^A methylation in a bidirectional manner. In particular, changes in m^6^A RNA methylation involving pathways related to neuronal function show reduced m^6^A RNA methylation and mRNA levels, consistent with synaptodendritic injury, neurodegenerative processes and reduced trophic support; conversely, pathways relevant to inflammatory processes and apoptosis show increased m^6^A RNA methylation and mRNA levels. Such direct correlation of m^6^A RNA methylation and mRNA levels in pathways involved in neurodegenerative and inflammatory processes is in keeping with the notion that m^6^A methylation promotes gene expression [21], and thus may serve to amplify transcriptional control of gene expression. Interestingly, we found changes in m^6^A methylation of several pathways related to RNA processing and metabolism to be negatively correlated with mRNA levels. m^6^A methylation has been shown to modulate several aspects of RNA metabolism [10, 63].

The present data suggest the regulation of genes involved in RNA processing as a possible new mechanism by which m^6^A RNA methylation can indirectly regulate RNA metabolism. The mechanisms through which m^6^A methylation regulates RNA processing and metabolism remain largely unclear. Interestingly, we did not observe significant differential expression of transcripts directly involved in the regulation of m^6^A methylation. This may indicate that small changes in levels of proteins involved in RNA modifications, or changes in their translational efficiency effect changes in the regulation of m^6^A RNA methylation *in vivo*. The present results also showed a considerable number of transcripts with differential m^6^A methylation that were not differentially expressed. Since m^6^A RNA methylation appears to mostly increase gene expression efficiency, these transcripts with increased m^6^A methylation but normal RNA levels may be mRNAs that are poised for greater induction or down-regulation should external stimuli result in activation or inhibition of their promoters. Alternatively, they may represent mRNAs with gene expression regulation at the translational level [9, 21].

The available evidence provides support for bidirectional regulation of m^6^A modification of mRNA. Addition and removal of m^6^A residues has been suggested to be dynamic, e.g. [14, 15]. This implies that modifications of RNA m^6^A content act as a separate layer of RNA regulation. Other evidence has suggested that m^6^A methylation occurs at the pre-mRNA level during transcription [16]. This scenario is consistent with coordinated differential expression and m^6^A modification of nascent mRNAs. Studies reported a direct correlation between m^6^A methylation and expression of both viral [43] and host proteins [1, 21]. It has been suggested that the coordinated actions of m^6^A writers, erasers and readers result in accelerating RNA processing and mRNA transport and result in more efficient but perhaps more transient translation [9, 21]. The m^6^A reader YTHDF1 in HeLa cells has been shown to localize mRNA to translation machinery and enhance translation efficiency [21]. Conversely the m^6^A reader YTHDF2 has been shown to localize m^6^A-methylated mRNA decay sites and reduce transcript stability [20]. Since m^6^A readers can play differential roles, it can be expected that the cellular and molecular context is key in determining the consequences of differential m^6^A methylation. Interaction of YTH domain ‘‘reader” proteins also regulates the efficiency of m^6^A methylated RNA processing and translation [20, 21]. However, it is important to note that these conclusions are based in part on studies of overexpression in cell lines and therefore it is possible that different results may be observed at different ratios of the proteins involved in m^6^A RNA methylation to mRNA and/or in different cell types. Changes in mRNA levels reflect the difference between transcription and degradation rates. Our results seem to indicate a role for m^6^A methylation in regulating mRNA levels in concert with transcriptional regulation as we observed a primarily positive correlation of the cellular processes affected by differential m^6^A methylation and differential gene expression.

A limitation of the present study is that the present results do not allow us to determine at what stage in the life of RNA is m^6^A methylation regulated or if it is the result of a dynamic process. Another limitation of the present study is that here we explored the occurrence and regulation of the most abundant of the RNA modifications of m^6^A, while several other chemical marks can occur and their elucidation may reveal an epitranscriptome “code” that directs RNA processing, metabolism and expression [6–9]. Additionally, the antibody used in the present study, while highly specific, does not distinguish m^6^A from the highly similar m^6^Am modifications, which also contain a 2’-O-methyl in the ribose moiety [64] and whose function is less characterized.

In conclusion, we used a m^6^A-directed antibody in conjunction with RNA-Seq to explore RNA m^6^A methylation *in vivo* in HIV Tg rats. The density of m^6^A in hippocampal mRNAs *in vivo* was greater in the distal CDS and 3’UTR and the greatest density was in the immediate vicinity of the stop codon. The pattern of m^6^A methylation in HIV transcripts in HIV Tg rats overall resembled the ones reported in cell lines and T cells *in vitro*. The function of the host genes and pathways affected by changes in m^6^A in HIV Tg rats was reminiscent of the pathways differentially regulated in both HIV Tg rats and humans with HIV in our previous studies [52, 53]. These include pathways involved in neural function, suggestive of synaptodendritic injury and neurodegeneration, immune response and inflammation, and, interestingly, RNA splicing and processing. Correlation of m^6^A RNA methylation and differential expression indicates that RNA methylation is a significant contributor to gene expression regulation in neuroAIDS in a bidirectional manner with pathway-specific differential regulation in different categories of genes. Thus, sets of transcripts enriched in m^6^A appear to be involved in coordinated transcriptional host responses in the context of chronic HIV. Overall our results support that m^6^A methylation is a mechanism for widespread regulation of mRNA *in vivo* that affects both HIV transcripts and host genes orchestrating host adaptive and maladaptive transcriptional effects of chronic HIV.

## Materials and methods

### Animals

Adult male HIV Tg rats in Sprague Dawley background (Harlan Sprague-Dawley) 5–6 months of age were housed in groups of two per cage in a temperature-controlled (20–26 ^o^C) vivarium on a 12h/12h light/dark cycle with ad libitum access to food and water. Rats were sacrificed by decapitation under deep CO_2_ anesthesia. All procedures adhered to the Guide for the Care and Use of Laboratory Animals and were approved by the Institutional Animal Care and Use Committee of The Scripps Research Institute.

### mRNA isolation and m^6^A-modified RNA immunoprecipitation (MeRNA-IP)

Total RNA was extracted from dissected hippocampi of the wildtype and HIV Tg rats with RNEasy kit (Qiagen) and poly-A selected with Dynabeads Oligo(dT)25 (Thermo Fisher Scientific) following manufacturer’s instructions. Poly-A enriched RNA was recovered by ethanol precipitation overnight, fragmented using RNA Fragmentation Reagents (Thermo Fisher Scientific) and ethanol precipitation overnight. An aliquot of fragmented RNA was set aside serving as input or control for RNA sequencing. Twenty μg of fragmented RNAs were denatured at 75°C for 5 min, cooled on ice for 3 min, and then incubated for 2h at 4°C with 3 μg of pre-equilibrated anti-m^6^A (Synaptic Systems), which was pre-immobilized to M-270 Epoxy using the Dynabeads Antibody Coupling kit (Thermo Fisher Scientific) for easy capture of the m^6^A-modified RNA in subsequent elution with a Proteinase K-containing buffer. Eluate with the anti-m^6^A captured RNA was extracted by phenol/CHCl3 followed by ethanol precipitation.

### RNA sequencing

Libraries for the anti-m6A-captured and control (fragmented, non-immunoprecipitated) RNAs were generated for next gen sequencing, following the protocol recommended in the ScriptSeq v2 RNA-Seq Library Preparation Kit (Illumina), and uniquely barcoded with the ScriptSeq Index PCR Primers (Illumina) to run a 16-library sample multiplex in a single flowcell on a NextSeq500 platform (Illumina) to generate ~30M 1x75 bp reads/sample.

### Data analysis

#### Reads pre-processing and mapping

Sequences were first trimmed using Trimmomatic with default setting (version 0.33). All the samples passed the quality control by fastQC (version 0.11.3). Fastq files were aligned to a combined rat genome (Rnor_6.0) and HIV-1 genome (NC_001802) using STAR (v2.4) with default setting. The overall mapping rate range from 79.78% to 85.79% (S3 Table)

#### Detecting RNA methylation sites

RNA methylation sites were detected using exomePeak (R package, version 2.10.0) in HIV infected rat samples and control samples separately. Aligned bam files of m6A treated samples and non-treated samples were directly passed to exomePeak to test for statistically significant enrichment. A total of 9696 and 9051 consist RNA methylation sites with at least 100 bp were identified in HIV infected samples and control samples respectively (p-value < = 0.05, S4 Table). For the mapping of the distribution of m^6^A peaks within host mRNAs, per base coverage was generated for all the m^6^A treated samples using R package “RiboProfiling” for a fixed interval of 400 bases around the start codon and stop codon.

#### Identifying genes with differential methylation and expression in HIV Tg rats vs. wild-type control rats

RNA-seq counts of 6,757 genes with at least one methylation site identified in the previous section *Detecting RNA Methylation Sites* were retrieved from m6A treated HIV infected samples and m6A treated controls using htseqcount2 with union mode. The rat genome was first humanized using ‘biomaRt’ package from R, only the gene with the highest counts were kept when multiple rat genes matched to one human gene. We then kept only the genes with an average raw count greater than 10. Differential methylation analysis was then performed using ‘DESeq2’ package in R (Wald method was used). 201 genes were identified with significant changes of methylation (p-value < 0.05) (S1 Table). Differential expression analysis was performed in a similar fashion for a total of 13,114 genes, where 383 genes were identified with significant differential expression (p-value < 0.01) (S5 Table). A set of 24 genes was both significantly modified in expression and methylation (S6 Table).

#### Identifying gene sets with enriched methylation and expression in HIV Tg rats vs. wild-type control rats

A total of 1,452 well-known gene sets from the MSigDB collection was tested with the GSEA algorithm. 166 gene sets were significantly changed in expression (p-value<0.05, 76 genes sets were up regulated in HIV infected samples while 90 gene sets were down regulated) (S7 Table) and 74 gene sets were significantly changed in methylation (p-value<0.05, 44 gene sets were up regulated in HIV infected samples while 30 gene sets were down regulated) (S2 Table). Among these gene sets, 6 were identified as significantly down regulated both at the level of expression and methylation while 9 were up regulated. The top 40 most enriched genes set are shown in Fig 3.

## Supporting information

S1 Table Genes with significant differential methylation. (XLSX)

S2 Table GSEA analysis of RNA methylation. (XLSX)

S3 Table Mapping quality. (XLSX)

S4 Table Genome region with enriched methylation identified by exomepeak. (XLSX)

S5 Table Genes with differential expression. (XLSX)

S6 Table Genes with both significant change in methylation and expression. (XLSX)

S7 Table GSEA analysis of gene expression. (XLSX)

S1 Fig RT-PCR of genes involved in RNA Methylation in the hippocampus of HIV Tg and wild-type rats. (DOCX)
