# Supplementary material for: Epitranscriptomics: Correlation of N6-methyladenosine RNA methylation and pathway dysregulation in the hippocampus of HIV transgenic rats
Source: PLoS One. 2019 Jan 17;14(1):e0203566. doi: 10.1371/journal.pone.0203566 (PMC6336335; doi:10.1371/journal.pone.0203566)
Supplement: S1 Fig — (DOCX) [file pone.0203566.s008.docx]

**
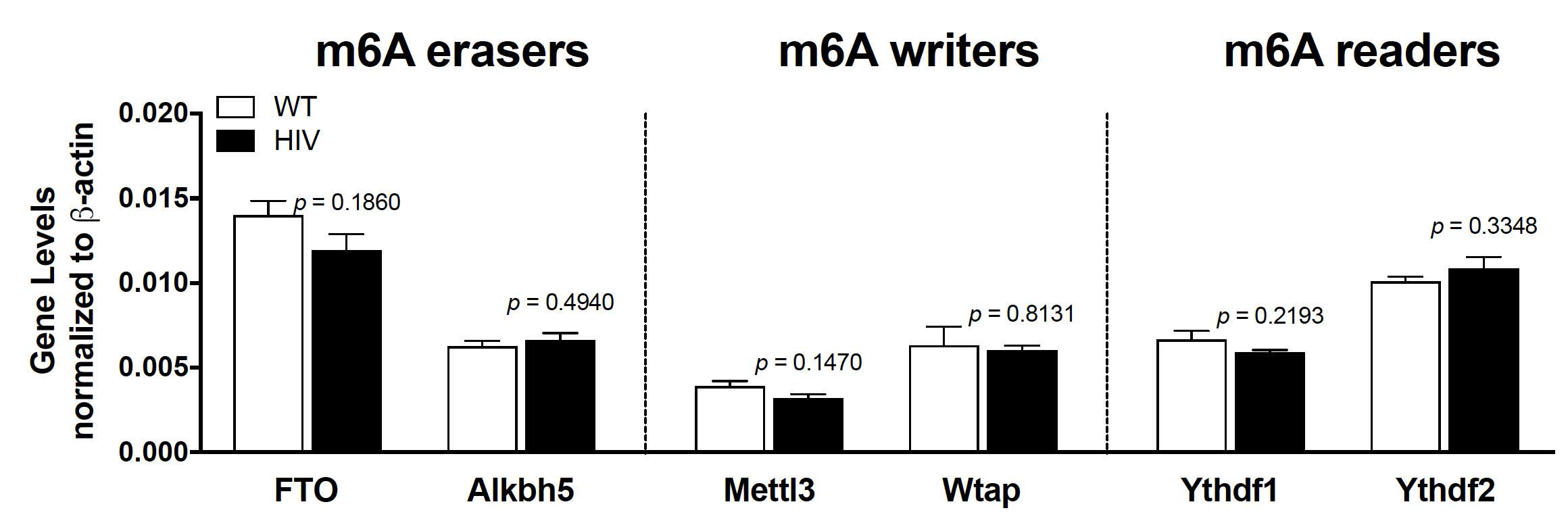
**

**Supplementary Fig. 1** RT-PCR of main N6-methyladenosine RNA methylation modifying enzymes in the hippocampus of HIV transgenic (HIV) and control rats (WT).
